# Giant isotropic negative thermal expansion in Y-doped samarium monosulfides by intra-atomic charge transfer

**DOI:** 10.1038/s41598-018-36568-w

**Published:** 2019-01-15

**Authors:** Koshi Takenaka, Daigo Asai, Ryoichi Kaizu, Yosuke Mizuno, Yasunori Yokoyama, Yoshihiko Okamoto, Naoyuki Katayama, Hiroyuki S. Suzuki, Yasutaka Imanaka

**Affiliations:** 1 Department of Applied Physics, Nagoya University, Furo-cho, Chikusa-ku, Nagoya, 464-8603 Japan; 2 Research Center for Advanced Measurement and Characterization, National Institute for Materials Science (NIMS), Sengen, Tsukuba, 305-0047 Japan; 3 Tsukuba Magnet Laboratory, National Institute for Materials Science (NIMS), Sakura, Tsukuba, 305-0003 Japan

## Abstract

Stimulated by strong demand for thermal expansion control from advanced modern industries, various giant negative thermal expansion (NTE) materials have been developed during the last decade. Nevertheless, most such materials exhibit anisotropic thermal expansion in the crystal lattice. Therefore, strains and cracks induced during repeated thermal cycling degrade their performance as thermal-expansion compensators. Here we achieved giant *isotropic* NTE with volume change exceeding 3%, up to 4.1%, via control of the electronic configuration in Sm atoms of SmS, (4 *f*)^6^ or (4 *f*)^5^(5*d*)^1^, by partial replacement of Sm with Y. Contrary to NTE originating from cooperative phenomena such as magnetism, the present NTE attributable to the *intra-atomic* phenomenon avoids the size effect of NTE and therefore provides us with fine-grained thermal-expansion compensators, which are strongly desired to control thermal expansion of microregions such as underfill of a three-dimensional integrated circuit. Volume control of lanthanide monosulfides via tuning of the 4 *f* electronic configuration presents avenues for novel mechanical functions of a material, such as a *volume-change* driven actuator by an electrical field, which has a different drive principle from those of conventional strain-driven actuators such as piezostrictive or magnetostrictive materials.

## Introduction

Successive discoveries of giant negative thermal expansion (NTE) materials during the last decade are causing a paradigm shift in the field of thermal expansion control^1,2^. Approaches using phase transitions^3–11^ achieved negative coefficients of linear thermal expansion (negative α) that are several times to ten times as large as those of conventional materials^12–16^, in which NTE originates from a characteristic of the crystal structure. Recently, the largest total volume change related to NTE, Δ*V*/*V* = 8.5%, was reported for Pb_0.76_La_0.04_Bi_0.20_VO_3_^11^. Extremely high NTE of α = −115 × 10^−6^ K^−1^ and total volume change Δ*V*/*V* = 6.7% are also realized in layered ruthenium oxides by the microstructural effects of a sintered body^17^.

These giant NTE materials enable us to compensate the thermal expansion of plastics^18–21^, which has been difficult to date. The technology of thermal expansion control is now being innovated. However, these “phase-transition-type” giant NTE materials are mostly anisotropic. Therefore, strains and defects are induced in the materials during repeated thermal cycling, thereby degrading the reproducibility of NTE functions and mechanical strength. To avoid these difficulties, *isotropic* NTE materials are strongly desired. A promising candidate is samarium monosulfide SmS, which exhibits large volume change exceeding 7% according to the 4 *f* electronic configuration in the Sm atom^22^. Two electronic configurations of Sm in SmS, (4 *f*)^6^ and (4 *f*)^5^(5*d*)^1^, compete energetically. At ambient pressure, the former configuration is more stable and the system is in the larger volume insulating state in black color (*black phase*). However, at the low pressure of 6 kbar, the latter configuration becomes more stable and the system switches to the smaller volume metallic state with golden color (*golden phase*)^23,24^. The cubic rock salt structure is preserved in this phase transition. Actually, this phase transition can be induced also by partial replacement of Sm by other elements. The smaller-volume golden phase appears at the higher temperature (*T*) side^25–30^. Results of an earlier x-ray diffraction (XRD) study suggest that volume contraction on heating reaches 3% in the case of Sm_1−*x*_Y_*x*_S^25^.

Samarium monosulfide has a long history of basic physics research related to its valence fluctuation^23–31^. Recently it has attracted great attention from the viewpoint of an excitonic insulator^32^ and a heavy-fermion metamaterial^33^. Nevertheless, few studies have examined it as a functional material showing NTE. The potential of a thermal-expansion compensator has yet been addressed only scarcely. We explored the thermal expansion properties of single-crystalline Sm_1−*x*_Y_*x*_S using XRD and dilatometry measurements. The present results revealed details of the compositional dependence of NTE in this solid-solution system and shed light on the peculiar mechanism of broadening volume change relevant to NTE. This report presents discussion of the rich potential possessed by the *intra-atomic charge transfer*, which is proposed as a volume-control principle for novel mechanical functions of materials.

## Results

**Figure 1:**
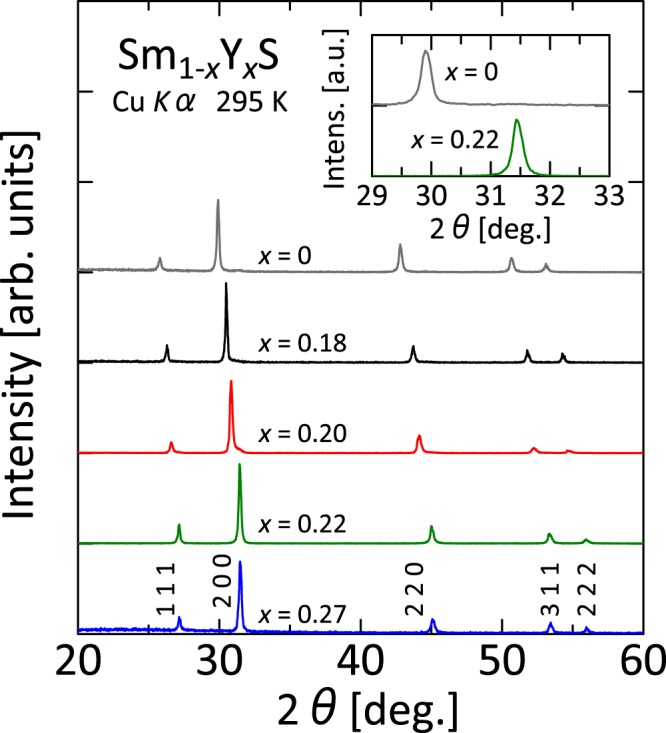
X-ray diffraction analyses of Sm_1−*x*_Y_*x*_S. X-ray diffraction profiles of Sm_1−*x*_Y_*x*_S using Cu *K*α radiation. The data were collected at room temperature (295 K). No extra peaks related to impurities were detected. Inset shows magnified profiles of the 200 peak for *x* = 0 and 0.22.

Substitution of Y for Sm induces the phase transition from a larger-volume black phase to a smaller-volume golden phase at ambient pressure^25–30^. This transition was confirmed also from results of the present study. Figure 1 portrays the XRD pattern of Sm_1−*x*_Y_*x*_S measured at room temperature (295 K) using Cu *K*α radiation. For all Y concentrations, no peaks attributable to impurities are detected. All the observed peaks can be indexed based on its cubic rock salt structure with *Fm*$\bar{3}$*m* symmetry. The inset is an enlarged figure of the 200 peak for *x* = 0 and 0.22. The peak widths are almost identical in doped and non-doped SmS, indicating that Sm and Y atoms are dispersed homogeneously. From *x* = 0.20 to *x* = 0.22, peak positions shift rapidly to higher angles, suggesting that the system undergoes the phase transition to the smaller-volume golden phase at 295 K and ambient pressure. The phase boundary is almost identical to the results described above.

**Figure 2:**
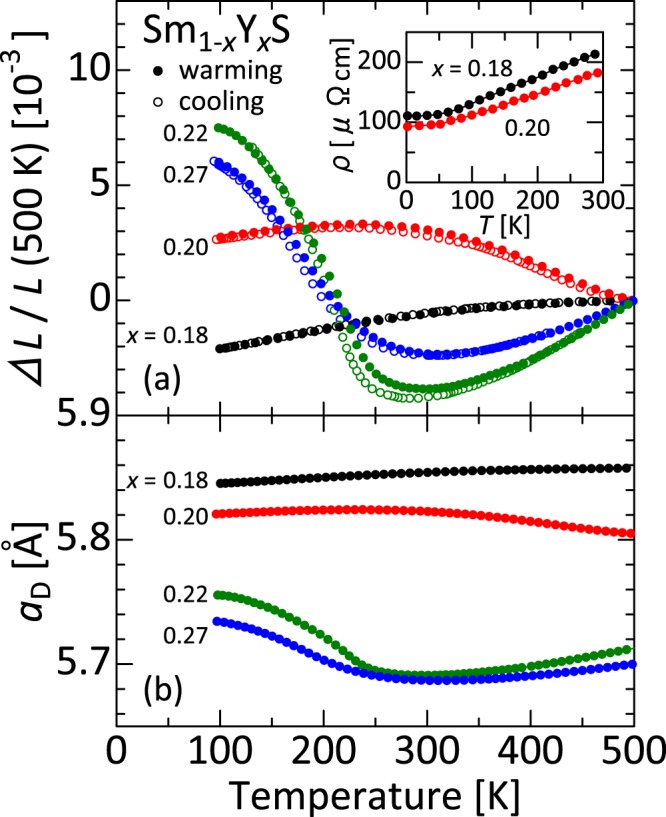
Thermal expansion properties of Sm_1−*x*_Y_*x*_S. (**a**) Linear thermal expansion Δ*L/L* of Sm_1−*x*_Y_*x*_S single crystals (reference temperature: 500 K). Data were collected on warming (solid circles) and cooling (open circles) processes using a laser interference dilatometer. Inset: Temperature dependence of resistivity ρ of Sm_1−*x*_Y_*x*_S. (**b**) “Dilatometric” lattice parameters, *a*_D_, estimated from the room-temperature x-ray diffraction (Fig. 1) and dilatometry (**a**) measurements, assuming that the cubic crystal structure is preserved in the whole temperature range.

Figure 2a displays linear thermal expansion Δ*L*(*T*)/*L* of single-crystalline Sm_1−*x*_Y_*x*_S. Negative thermal expansion corresponds to the transition from black to golden phase. Solid and open circles respectively represent data collected for warming and cooling processes. In general, hysteresis is not remarkable, although it is about Δ*T* = 10 K at maximum for *x* = 0.22. Below the onset temperature *T*^on^ = 290 K, giant NTE of α = −60 × 10^−6^ K^−1^ appears for *x* = 0.22. The total volume change related to NTE, Δ*V*/*V*, reaches 3.4% at 100−290 K, which is comparable to the lattice-parameter change exceeding 1% between 140 K and 375 K estimated from the previous XRD analysis for *x* = 0.25^25^. Among isotropic NTE materials, the present volume change is the largest: much greater than the former record of 2.7% in ZrW_2_O_8_^12^ (Table 1). Additionally, it is noteworthy that the operating-temperature window is exceptionally wide (Δ*T* >190 K) for phase-transition-type NTE materials. Reports of earlier studies have described that the system becomes metallic immediately by Y doping^30^. The present resistivity ρ(*T*) data shown in the inset of Fig. 2a are consistent with data found in earlier works.

**Table 1:** Parameters related to negative thermal expansion for prototypical isotropic giant negative thermal expansion materials.

	Δ*V*/*V* [%]	*T*_NTE_ [K]	Δ*T* [K]	α[ppm/K]	Method^a^	Reference
ZrW_2_O_8_	2.7	2–1443	1441	−6~−9	D/N	^12^
Cd(CN)_2_·*x*CCl_4_	2.1	170–375	205	−34	X	^14^
ScF_3_	0.6	150–425	275	−7	X	^16^
Mn_3_Ga_0.7_Ge_0.3_N_0.88_C_0.12_	0.5	197–319	122	−18	D	^3^
LaFe_10.5_Co_1.0_Si_1.5_	1.1	240–350	110	−26	D	^6^
Sm_0.78_Y_0.22_S	4.1	100–315	215	−65	D	this work

^a^D, dilatometry; N, neutron diffraction; X, x-ray diffraction.

**Figure 3:**
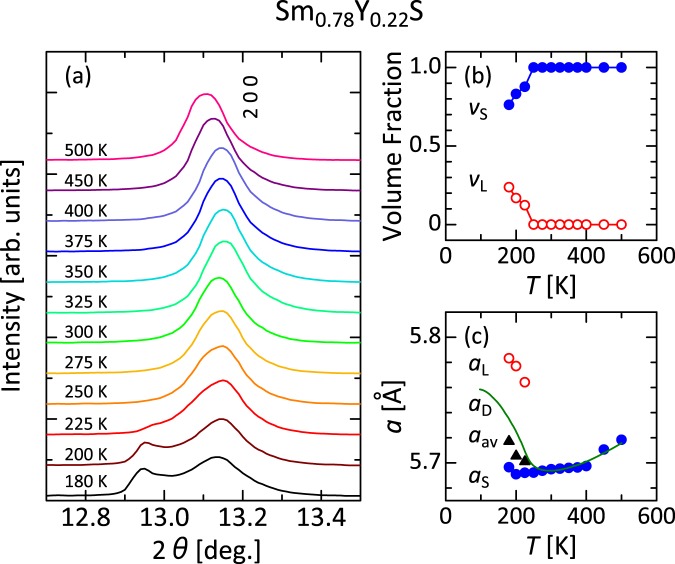
Temperature dependence of the crystallographic parameters. The parameters are ascertained from Rietveld analysis results (see Supplementary Fig. S1) (**a**) Temperature dependence of the 200 peak in the x-ray diffraction measurements for Sm_0.78_Y_0.22_S using synchrotron radiation (λ = 0.6521 Å). (**b**) Fraction of the larger-volume L and smaller-volume S phases: *v*_L_ and *v*_S_. (**c**) Lattice parameter of the L and S phases: *a*_L_ and *a*_S_. The averaged lattice parameter *a*_av_ = *v*_L_*a*_L_ + *v*_S_*a*_S_, and the “dilatometric” lattice parameter *a*_D_, presented in Fig. 2b, are also shown.

We analyzed the crystal structures of Sm_0.78_Y_0.22_S using synchrotron x-ray diffractometry to ascertain how the crystal lattice changes related to the giant NTE (Fig. 3a). The structural parameters are refined using the Rietveld method by RIETAN-FP^34^ (Supplementary Fig. S1). At *T* = 250−500 K, the system preserved the single phase of cubic rock salt structure with *Fm*$\bar{3}$*m* symmetry. However, at temperatures below 225 K, it is apparently separated into two peaks. Therefore, we conducted a Rietveld analysis assuming two phases with the same cubic crystal structure but a different lattice constant, i.e., the larger-volume L phase and the smaller-volume S phase. The higher-*T* diffraction data above 250 K are continuous with the S phase. Each phase preserves the same cubic structure of *Fm*$\bar{3}$*m* symmetry. Figure 3b displays the respective *T*-dependent fractions of the L and S phases obtained by Rietveld analysis: *v*_L_ and *v*_S_. At temperatures higher than 250 K, Rietveld analysis assuming a single phase was conducted (*v*_L_ = 0 and *v*_S_ = 1) because no significant difference was found between analysis assuming two-phase coexistence and that assuming a single phase. At temperatures lower than 225 K, *v*_L_ increases concomitantly with decreasing *T*. Figure 3c displays the respective lattice parameters of the L and S phases: *a*_L_ and *a*_S_. *a*_L_ increases concomitantly with decreasing *T* (*i.e., a*_L_ shows NTE). It elongates to 5.7832(2) Å at 180 K, which is 1.6% elongation compared with the lattice parameter at 300 K (just above *T*^on^ = 290 K), 5.6949(2) Å. However, *a*_S_ decreases continuously, despite the phase separation, with decreasing *T* down to 200 K. Impressively, it turns upward with further cooling, increasing from 5.6909(2) Å at 200 K to 5.6965(2) Å at 180 K (*i.e., a*_S_ shows NTE at *T* = 180−200 K). Figure 3c displays *a*_L,_ *a*_S_, and their averaged sum, *a*_av_ = *v*_L_*a*_L + _*v*_S_*a*_S_. At 200 K, for example, *a*_L_ = 5.6909(2) Å, *a*_S_ = 5.7770(3) Å, *v*_L_ = 0.831, and *v*_S_ = 0.169. As a result, *a*_av_ is calculated to be 5.705 Å. For comparison, it also shows the “dilatometric” lattice parameter *a*_D_ (Fig. 2b), estimated based on dilatometry data and the room-temperature (295 K) lattice parameter, assuming that the cubic structure is preserved over the whole *T* region. Here *a*_av_ = *a*_S_ at *T* = 250−500 K because *v*_L_ is zero in this region. *a*_av_ is almost identical to *a*_D_, implying that NTE of the bulk crystal originates from NTE of crystallographic unit cells. It differs from the giant NTE in Ca_2_RuO_4_ ceramics, in which microstructural effects in a sintered body play an important role^17,21^. We also conducted Rietveld analysis by assuming three phases (Supplementary Fig. S2). The reliability factor improves from *R*_wp_ = 2.76%, *R*_p_ = 2.22% and *S* = 1.25 for two phase analysis to *R*_wp_ = 2.60%, *R*_p_ = 2.09% and *S* = 1.17 by assuming three phases, but it is natural because the fitting parameters increase. Rather, probably two-phase analysis is fairly good as long as the difference is of this order of magnitude. In the three-phase analysis, the coincidence between *a*_av_ and *a*_D_ improves. In this case *a*_av_ is defined to be Σ*v*_*i*_*a*_*i*_ (*v*_*i*_ and *a*_*i*_ being respectively the fraction and the lattice parameter of the phase *i* obtained by Rietveld analysis and Σ*v*_*i*_ = 1). Therefore, the core part of the arguments – anomaly in the crystallographic unit-cell volume is relevant to NTE – is not change even if we adopt a three-phase analysis.

Phase-transition-type NTE materials are broadly divisible into two categories: “phase-separation” type and “second-order-transition” type. In the former category, which includes bismuth-nickel oxides^9,19^, the system separates into the larger-volume L phase and the smaller-volume S phase, while preserving the first-order phase transition. NTE is induced because the fraction of the L phase increases concomitantly with decreasing *T*. In that case, it is important that no phase exhibit NTE. This class of phase-transition-type NTE materials is, so to speak, in a state to be described as “homocomposite”, in the sense that it is divided into domains with different physical properties such as lattice volume though chemically homogeneous. In the latter category, which includes magnetic NTE materials such as antiperovskite manganese nitrides^3^, the phase transition changes from first-order-like to second-order-like. The volume increases gradually with decreasing *T* without phase separation. It is noteworthy that Sm_1−*x*_Y_*x*_S has *both* characters. Although the large NTE of Sm_1−*x*_Y_*x*_S is dominated by phase separation between the L and S phases, each phase shows NTE below a certain temperature. The smaller hysteresis in Sm_1−*x*_Y_*x*_S than in the bismuth-nickel oxides^19^ is partly explainable by this duality. The peculiarity of the phase-separation behavior is later discussed.

Figure 2b presents the *T* and Y-concentration dependence of *a*_D_ for Sm_1−*x*_Y_*x*_S. This result is reminiscent of past findings related to Y-concentration dependence of the boundary temperature T^*^ between the black and golden phases in Sm_1−*x*_Y_*x*_S^26^. In that study, the golden phase suddenly appears at around *x* = 0.15; the T^*^(*x*) curve is almost vertical there. Actually, T^*^(*x*), which is highest (600 K) at around *x* = 0.15, instantly decreases to 250 K by further Y doping, and subsequently decreases slowly, concomitantly with increasing *x*. It becomes about 200 K for *x* = 0.3. Systems are, respectively, in the golden and black phases above and below T^*^. Past x-ray absorption studies have indicated that this black-to-golden phase transition results from intra-atomic charge transfer from 4 *f* to 5*d* orbitals in Sm atom^29^. In the present experiments, the onset temperature of NTE, *T*^on^, exceeds 500 K for *x* = 0.20, decreases rapidly to 290 K for *x* = 0.22, and decreases slowly for additional Y doping. Present correlations between T^*^ and *T*^on^ imply that the giant NTE observed in this study originates from the lattice volume change because of the intra-atomic charge transfer in Sm atoms.

## Discussion

The unit-cell volume of SmS is related closely to the number of 4 *f* electrons in a Sm atom. Yttrium is a useful dopant for tuning the Sm 4 *f* electronic states in SmS. However, a Y atom in Sm_1−*x*_Y_*x*_S donates one electron to the *d* band. In addition, the 4 *f* electronic state is apparently quite sensitive to Y.

Additional electrons donated by Y dopants might prevent us from realizing the full charge transfer related to NTE in SmS. In the case of pressure-induced golden phase, the charge transfer in Sm is almost one [(4 *f*)^6^ → (4 *f*)^5^]^24^. This charge transfer induces large volume change up to 7%. However, the intra-atomic charge transfer caused by temperature in Sm_1−*x*_Y_*x*_S is much smaller. An earlier x-ray absorption study revealed that the charge transfer from 10 K to 300 K as 0.13 [(4 *f*)^5.65^ → (4 *f*)^5.52^] for Sm_0.67_Y_0.33_S^29^. This might be true presumably because the electronic state near the Fermi level is altered by the electrons donated by Y.

The excessive sensitivity of Y produces a wide blank region between *x* = 0.20 and *x* = 0.22 (Fig. 2b). As Y doping proceeds, the bottom of the 5*d* band lowers. Around this concentration, it fully overlaps with the 4 *f* level and the charge transfer from 4 *f* to 5*d* levels occurs intensely^25^. For Sm_0.80_Y_0.20_S, in which *T*^on^ exceeds 500 K, the black phase is yet stable. The volume does not contract so much, even at high temperatures, resulting in weak NTE (α = −12 × 10^−6^ K^−1^ at 230−500 K). However, a slight amount of additional Y dopants drastically alters the electronic state and the golden phase is stabilized. As a result, for *x* = 0.22, *T*^on^ decreases to 290 K. The volume does not expand greatly, even at low *T*, compared to *x* = 0.20.

In addition, the excessive sensitivity induces phase separation in the critical composition *x* = 0.22. In general, inhomogeneity in atomic scale is intrinsically unavoidable in a solid solution no matter how high the quality to which we form it. The Sm atom in Sm_1−*x*_Y_*x*_S has twelve nearest-neighbor Sm(Y) atoms and these twelve sites are randomly occupied by Sm with a probability of 0.78 and by Y with a probability of 0.22 for *x* = 0.22, for example. It is important that in the present system such an atomic fluctuation may produce a large difference in lattice parameter because of the sensitivity. The phase separation in the present XRD analysis reflects this peculiarity of Sm_1−*x*_Y_*x*_S, which might be qualitatively different from the usual chemical phase separation. This unique phase separation phenomenon is a difficulty that is related directly to improvement of NTE properties. This study shed light on important subjects to pursue as possibilities of this monosulfide for use as a thermal-expansion compensator. The phase separation phenomena should be explored in detail in future studies.

**Figure 4:**
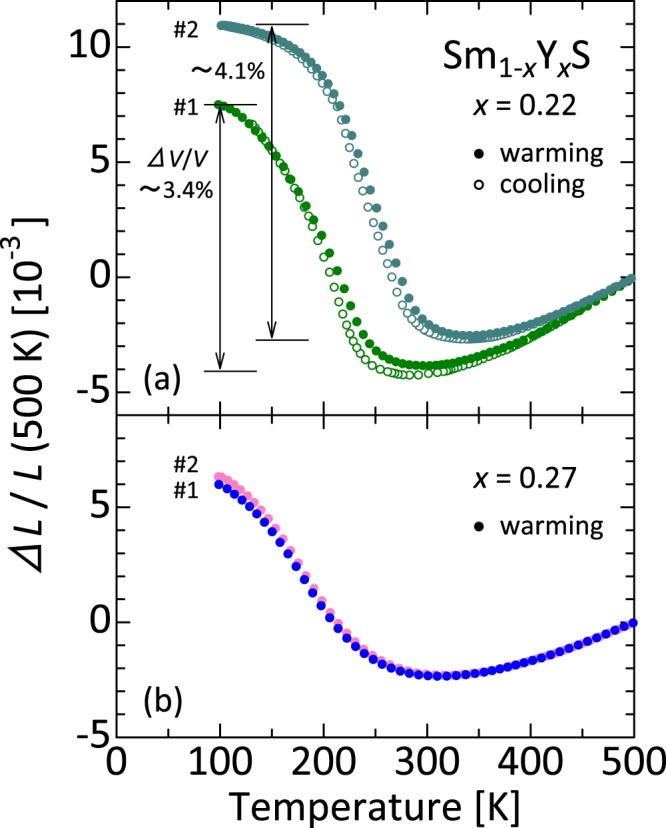
Sample dependence of linear thermal expansion for Sm_1−*x*_Y_*x*_S. (**a**) *x* = 0.22 and (**b**) *x* = 0.27 (reference temperature: 500 K). For each composition, the data labeled as #1 are also presented in Fig. 2a. There exists sample dependence in the thermal expansion data for the critical composition *x* = 0.22, although the data are reproducible for *x* = 0.27. We found the largest total volume change related to negative thermal expansion, Δ*V*/*V* ~ 4.1%, for the crystal labeled as #2 for *x* = 0.22, but x-ray diffraction peaks of this crystal are multiply splitting at low temperatures (see Supplementary Fig. S3), which suggests that the lattice parameter is highly fluctuating within the crystal.

We have experimentally obtained results predicting that optimization of tuning of the Sm 4 *f* electronic state engenders higher performance of the NTE function. In the critical composition *x* = 0.22, sample dependence was observed in the dilatometry result. A crystal showing a larger total volume change than the one discussed above was also found (Fig. 4a). In this crystal, giant NTE of α = −65 × 10^−6^ K^−1^ appears below *T*^on^ = 315 K and Δ*V*/*V* reaches 4.1%, which is among the largest of all the NTE materials reported to date. Although the XRD pattern of this crystal measured at 295 K using Cu *K*α radiation is similar to that of #1 presented in Fig. 1, the pattern obtained from high-resolution synchrotron x-ray diffractometry clearly indicates the presence of multiple divided peaks (Supplementary Fig. S3). The XRD pattern can be refined roughly to obtain the averaged lattice parameter *a*_av_ by assuming multiple cubic phases with the same *Fm*$\bar{3}$*m* symmetry and different lattice parameters obtained by Rietveld analysis (*a*_av_ = Σ*v*_*i*_*a*_*i*_). Considering that the data reproducibility is good for *x* = 0.27 (Fig. 4b), the present result might be ascribed to sensitivity of the electronic state to Y in the critical composition rather than to a different degree of the chemical inhomogeneity.

Although fine tuning of the Sm 4 *f* electronic state might be difficult to achieve solely by Y doping, the samarium monosulfides have many routes to control the electronic states. Elements other than Y have also been regarded as dopants. To date, as a single dopant, Y is superior in terms of the operating temperature and the total volume change related to NTE Δ*V*/*V*^24,26^, but simultaneous doping of Y and other elements might solve this sensitivity difficulty. Another important avenue is greater use of the S site^35^. High-*T* crystal growth of SmS in principle might induce nonstoichiometry of sulfur. An earlier study we conducted using the same grade of the present crystals^30–33^ revealed that no fluctuation exists in the sulfur content that affects the systematic characteristics of the data. However, sulfur content and substitution of sulfur sites are important in the future tuning of NTE properties. Optimization of these parameters might fill in the blank region between *x* = 0.20 and *x* = 0.22 and provide a giant NTE with larger volume change and higher *T*^on^. The present experiment suggests that large volume change Δ*V/V* of 4.8% (equivalent to Δ*a*/*a* = 1.6%) is possible if the L phase can be purified (see the temperature dependence of *a*_L_ depicted in Fig. 3c).

Because it is an atomic phenomenon related to the 4 *f* electronic configuration of the Sm atom, NTE of the samarium monosulfides presents potential advantages over NTE of existing materials. First, isotropy can be an important property for a thermal-expansion compensator in the sense that NTE function is reproducible without defects or strains against repeated thermal cycling. At present, for the critical concentration around at *x* = 0.22 showing large NTE, some crystals are broken during repeated thermal cycling, which indicates that internal strain exists because of the effect of phase separation. The potential advantage of isotropy has not been realized yet. This tendency is not observed for the Y concentrations deviating from *x* = 0.22. Therefore, this difficulty originates from the sensitivity of the Y dopant. It should be solved by optimizing the 4 *f* electronic state using simultaneous doping of Y and other elements. Next, NTE originating from atomic phenomena is independent of the grain size. For that reason, the difficulty that NTE function depends strongly on the grain size, characteristics of manganese nitrides^36^, can be avoided. Recently, fine-grained thermal-expansion compensators with submicrometer grain size are sought for use in many fields of industry, including electronics^37^, to control the thermal expansion of microregions. Finally, the wide operating-temperature window of Sm_1−*x*_Y_*x*_S might be attributable to a characteristic of the atomic phenomena. For cooperative phenomena such as magnetic and charge orderings, it is difficult to make, artificially, the gradual phase transition, which is the cause of the narrow operating-temperature window of the phase-transition-type NTE materials. By contrast, the phase transition related to atomic phenomena is expected to be susceptible to external perturbation. Thereby, the operating-temperature window would be expanded.

Volume control of lanthanide monosulfides via tuning of the 4 *f* electronic configuration (intra-atomic charge transfer) opens avenues towards novel mechanical functions of a material. Although similar concepts have been suggested to date for Sm_2.75_C_60_^38^ and YbGaGe^39^, the monosulfides are absolutely attractive in terms of variation in materials and in terms of their physical properties^22^. For example, an electrical field might induce the metallic phase because the insulating black phase and the metallic golden phase compete energetically^40^. This field-induced transition might be accompanied by large volume change at largest 7%. These are useful as a *volume-change* driven actuator, which has a different drive principle from conventional strain-driven actuators such as piezostrictive or magnetostrictive materials.

## Methods

### Sample preparation

Single crystals of Sm_1−*x*_Y_*x*_S were grown using Bridgeman method^31^. Powders of Sm, Y, and S (99.9% or higher purity) weighed at appropriate molar ratios were mixed in a glove box and were sealed in a quartz tube under vacuum (<10^−3^ Pa). The quartz ampoule was heated at a temperature of *T* = 873 K for 6 h and was then cooled to 573 K for 24 h. The obtained powder was reground and was then reheated in the same condition. Finally, the obtained powder was reground and was sealed in a tungsten crucible (15 mm diameter, 75 mm long) under vacuum (<10^−3^ Pa) using an electron beam welding system. The sealed tungsten crucible was heated with an induction heating furnace up to 2453 K for 20 h, held for 2 h, then cooled to 2173 K for 24 h and cooled to 1073 K. Subsequently, the furnace was switched off. We analyzed the compositional ratio between samarium and yttrium using inductively coupled plasma (ICP) method. The obtained crystals were identified as the monosulfide from x-ray powder diffraction measurements at room temperature with Cu *K*α radiation (Rint2000; Rigaku Corp.).

### Measurements of physical properties

Detailed crystallographic analyses were conducted using synchrotron x-ray powder diffractomety at 180−500 K at Aichi Synchrotron Radiation Center with synchrotron radiation of λ = 0.6521 Å. Linear thermal expansion Δ*L*(*T*)/*L* was measured using a rectangular crystal (typically 5 × 5 × 12 mm^3^) by means of a laser-interference dilatometer (LIX-2; Ulvac, Inc.) at *T* = 100−500 K. Several specimens broke into pieces during repeated thermal cycling because of the large volume change. In such cases, we used *pressed powder without sintering* to measure the linear thermal expansion. For some compositions, we confirmed that the data obtained for the pressed samples are equivalent to those obtained for the single crystals.

## Electronic supplementary material

Supplementary Information

## Data Availability

Data supporting the findings of this study are available from the corresponding author on request.
