# Supplementary material for: Giant isotropic negative thermal expansion in Y-doped samarium monosulfides by intra-atomic charge transfer
Source: Sci Rep. 2019 Jan 15;9:122. doi: 10.1038/s41598-018-36568-w (PMC6333773; doi:10.1038/s41598-018-36568-w)
Supplement: Supplementary file 1 — Supplementary Information [file 41598_2018_36568_MOESM1_ESM.docx]

**Supplemantary Information**

**Giant isotropic negative thermal expansion in Y-doped samarium monosulfides by intra-atomic charge transfer**

Koshi Takenaka,^1, †^ Daigo Asai,^1^ Ryoichi Kaizu,^1^ Yosuke Mizuno,^1^ Yasunori Yokoyama,^1^ Yoshihiko Okamoto,^1^ Naoyuki Katayama,^1^ Hiroyuki S. Suzuki^2^ & Yasutaka Imanaka^3^

^1^ *Department of Applied Physics, Nagoya University, Furo-cho, Chikusa-ku, Nagoya 464-8603, Japan*

^2^ *Quantum Beam Center, National Institute for Materials Science (NIMS), Sengen, Tsukuba 305-0047, Japan*

^3^ *Tsukuba Magnet Laboratory, National Institute for Materials Science (NIMS), Sakura, Tsukuba 305-0003, Japan*

**Supplementary Figure S1 | Rietveld refinement of Sm_0.78_Y_0.22_S (#1) by assuming two phases.** The experimental (cross) and fitted (line) x-ray diffraction pattern at 500 K, 300 K, and 200 K are shown. Vartical bars under the diffraction peaks show the Bragg reflection positions. The plots under the bars represent residues. All of the observed peaks can be indexed based on the *Fmm* symmetry. Below 225 K, we assumed two phases: the larger-volume L phase and the smaller-volume S phase. Each phase preserves the same cubic structure of *Fmm* symmetry.

(a)


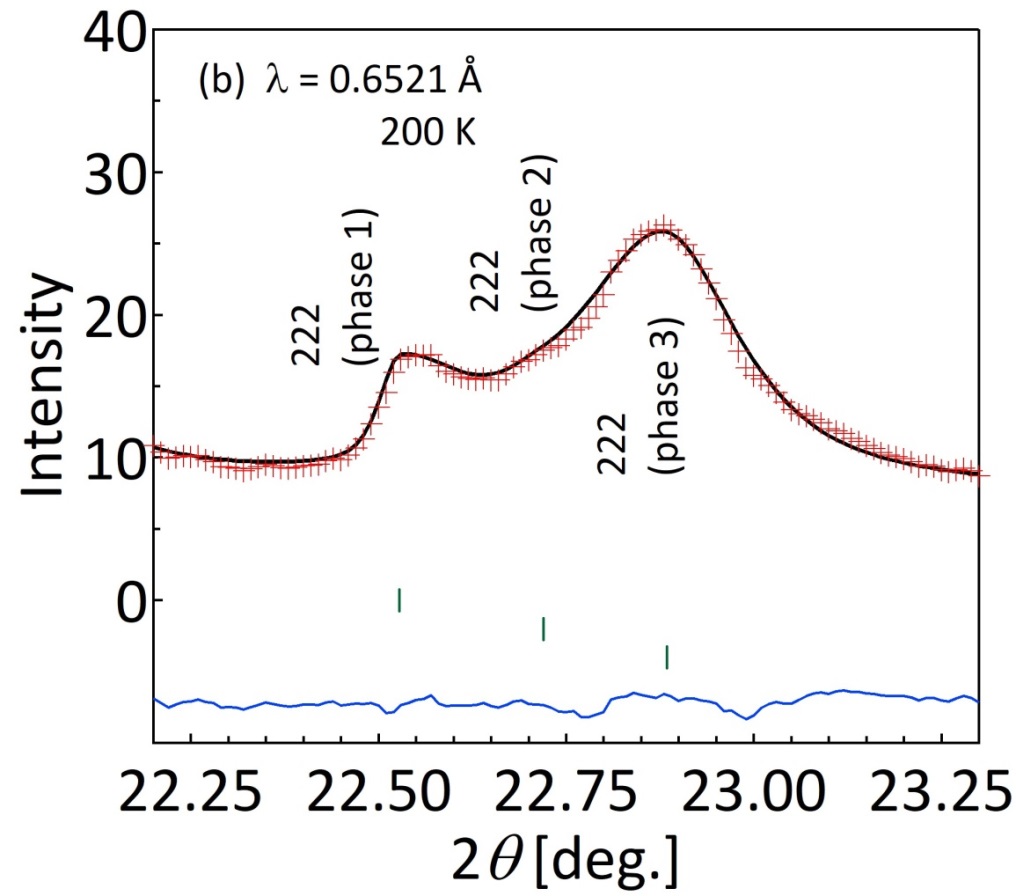


**Supplementary Figure S2 | Rietveld refinement of Sm_0.78_Y_0.22_S (#1) by assuming three phases.** (a) The experimental (cross) and fitted (line) x-ray diffraction pattern at 200 K are shown. Vartical bars under the diffraction peaks show the Bragg reflection positions. The plots under the bars represent residues. Each phase preserves the same cubic structure of *Fmm* symmetry. The averaged lattice parameter *a*_av_ (inset) is defined to be Σ*v_i_a_i_* (*v_i_* and *a*_i_ being respectively the fraction and the lattice parameter of the phase *i*, and Σ*v_i_*=1). At 200 K, *a*_1_=5.7785(3) Å, *a*_2_=5.7302(2) Å, *a*_3_=5.6898(2) Å, *v*_1_=0.158, *v*_2_=0.211, and *v*_3_=0.631. As a result, *a*_av_ is calculated to be 5.712 Å. (b) The 222 Bragg reflection positions of the three phases are shown.

**Supplementary Figure S3 | X-ray diffraction analyses of Sm_0.78_Y_0.22_S (#2).** (a) The x-ray diffraction (XRD) pattern was measured at 295 K using Cu *K*α radiation. No peak attributable to impurities was detected. All observed peaks can be indexed based on the cubic rock salt structure with *Fmm* symmetry. (b) The experimental (cross) and fitted (black line) high-resolution synchrotron XRD pattern are shown. Vartical bars under the diffraction peaks show the 222 Bragg reflection position of the four phases. The pattern revealed multiple peaks. The averaged lattice parameter *a*_av_ can be estimated roughly from Rietveld analysis by assuming four cubic phases with *Fmm* symmetry. Inset: The obtained *a*_av_ is fairly consistent with the dilatometry result, *a*_D_, suggesting that the present giant NTE originates from NTE of the crystallographic unit cell. At 220 K, *a*_1_=5.7549(1) Å, *a*_2_=5.7319(2) Å, *a*_3_=5.7138(1) Å, *a*_4_=5.6882(3) Å, *v*_1_=0.490, *v*_2_=0.060, *v*_3_=0.432, and *v*_4_=0.018. As a result, *a*_av_=Σ*v_i_a_i_* is calculated to be 5.735 Å.
